# Complex insight on microanatomy of larval “human broad tapeworm” *Dibothriocephalus latus* (Cestoda: Diphyllobothriidea)

**DOI:** 10.1186/s13071-019-3664-8

**Published:** 2019-08-21

**Authors:** Daniel Barčák, Aneta Yoneva, Hana Sehadová, Mikuláš Oros, Andrea Gustinelli, Roman Kuchta

**Affiliations:** 1 Institute of Parasitology, Slovak Academy of Sciences, Hlinkova 3, 04001 Košice, Slovak Republic; 2 Institute of Parasitology, Biology Centre of the Czech Academy of Sciences, Branišovská 31, 37005 České Budějovice, Czech Republic; 3 Institute of Biodiversity and Ecosystem Research, Bulgarian Academy of Sciences, 2 Gagarin Street, 1113 Sofia, Bulgaria; 4 Institute of Entomology, Biology Centre of the Czech Academy of Sciences, Branišovská 31, 37005 České Budějovice, Czech Republic; 5 Department of Veterinary Medical Sciences, University of Bologna, Via Tolara di Sopra 50, 40064 Ozzano Emilia, BO Italy; 6 University of South Bohemia, Faculty of Science, 37005 České Budějovice, Czech Republic

**Keywords:** Ultrastructure, Immunofluorescence, Plerocercoids, Microtriches, Receptors, Glands, Protonephridia, Cestoda

## Abstract

**Background:**

In Europe, the tapeworm *Dibothriocephalus latus* (syn. *Diphyllobothrium latum*) is a well-known etiological agent of human diphyllobothriosis, which spreads by the consumption of raw fish flesh infected by plerocercoids (tapeworm’s larval stage). However, the process of parasite establishment in both intermediate and definitive hosts is poorly understood. This study was targeted mainly on the scolex (anterior part) of the plerocercoid of this species, which facilitates penetration of the parasite in intermediate paratenic fish hosts, and subsequently its attachment to the intestine of the definitive host.

**Methods:**

Plerocercoids were isolated from the musculature of European perch (*Perca fluviatilis*) caught in Italian alpine lakes. Parasites were examined using confocal microscopy, scanning electron microscopy (SEM) and transmission electron microscopy (TEM). Immunofluorescence tagging was held on whole mount larvae.

**Results:**

The organisation of the central and peripheral nervous system was captured in *D. latus* plerocercoids, including the ultrastructure of the nerve cells possessing large dense neurosecretory granules. Two types of nerve fibres run from the body surface toward the nerve plexus located in the parenchyma on each side of bothria. One type of these fibres was found to be serotoninergic and possessed large subtegumental nerve cell bodies. A well-developed gland apparatus, found throughout the plerocercoid parenchyma, produced heterogeneous granules with lucent core packed in a dense layer. Three different types of microtriches occurred on the scolex and body surface of plerocercoids of *D. latus*: (i) uncinate spinitriches; (ii) coniform spinitriches; and (iii) capilliform filitriches. Non-ciliated sensory receptors were observed between the distal cytoplasm of the tegument and the underlying musculature.

**Conclusions:**

Confocal laser scanning microscopy and electron microscopy (SEM and TEM) showed the detailed microanatomy of the nervous system in the scolex of plerocercoids, and also several differences in the larval stages compared with adult *D. latus.* These features, i.e. well-developed glandular system and massive hook-shaped uncinate spinitriches, are thus probably required for plerocercoids inhabiting fish hosts and also for their post-infection attachment in the human intestine.

**Electronic supplementary material:**

The online version of this article (10.1186/s13071-019-3664-8) contains supplementary material, which is available to authorized users.

## Background

The tapeworm *Dibothriocephalus latus* (Linnaeus, 1758) (syn. *Diphyllobothrium latum*) is one of the causative agents of diphyllobothriosis with an estimated 20 million human cases worldwide [1–3]. Although the disease is often asymptomatic or manifests as general symptoms of mild abdominal discomfort, several dozen of clinical cases are annually reported in European countries [4]. The infectious stage is represented by plerocercoids inhabiting the musculature of several freshwater fishes including the European perch (*Perca fluviatilis* L.) [4]. The successful infection of definitive host is determined by the consumption of raw or undercooked fish flesh (e.g. “carpaccio di persico”, “sashimi” and others) and consequent attachment of the tapeworm in the host intestinal mucosa. To ensure the latter, the scolex possesses specialised muscular attachment organs, bothria, tiny projections of tegument, i.e. microtriches (for their terminology see Chervy [5]) and a complex of glandular cells (frontal glands), which release their secretory products of perhaps adhesive nature on the tegument [6]. The functional complexity of the scolex is determined by its rich innervation and by presence of distinct molecules identified as neurotransmitters, e.g. acetylcholin, peptides, serotonin (5-hydroxytryptamin, 5-HT) and synapsin [7, 8], whose functions are not sufficiently understood.

Most ultrastructural and immunochemistry/immunofluorescense-based studies of diphyllobothriidean plerocercoids have dealt with the congeneric species, *Dibothriocephalus dendriticus* (Nitzsch, 1824) [9–13], while few have investigated *D. latus* [14]. However, these two species differ in the morphology and life-cycle strategy of their larval stages (the former remain in the body cavity, while the latter migrate to the musculature), and in preferences for the second intermediate and definitive hosts [4]. Kuperman & Davydov [14] also stated that the glandular system of *D. latus* is much more developed in comparison with its congeners; this may coincide with the higher invasive potential of *D. latus* than that of *D. dendriticus* and *D. ditremus* (Creplin, 1925), as tested on paratenic hosts [15].

Here, an integrative approach combining confocal laser scanning microscopy (CLSM) with transmission electron microscopy (TEM) and scanning electron microscopy (SEM) was used to provide a more complex insight on the functional microanatomy of *D. latus* plerocercoids.

## Methods

### Parasite isolation

Plerocercoids of *Dibothriocephalus latus* occurred free in the musculature of naturally infected perch (*Perca fluviatilis*) captured in Lake Iseo and Lake Como, northern Italy, in April 2016 and 2017, respectively. They were immediately rinsed in 0.9% NaCl solution (TEM, SEM) or 0.1 M PBS solution (CLSM) at room temperature (≈ 20 °C) and processed by procedures as stated below.

### Confocal laser scanning microscopy (CLSM)

For immunofluorescence labelling, live larvae were fixed in two ways: (i) 6 individuals were fixed in fresh 4% formaldehyde solution at 4 °C (conventional processing), while (ii) 3 individuals were first treated by almost boiling 0.1 M PBS (heat-treatment), and consequently fixed in fresh 4% formaldehyde solution at 4 °C. After 24 h at 4 °C, all plerocercoids were removed from fixative, rinsed 3 times in 0.1 M PBS and stored in 0.1 M PBS with 0.03% sodium azide at 4 °C.

Immunolabeling of whole mount larvae (not sectioned) was initiated by their permeabilization in 0.1 M PB buffer with 0.5% Triton X (PBTrX), followed by incubation in 5% goat serum (GS) in PBTrX for 2 h. Microtubules were tagged by monoclonal anti-β tubulin antibodies (Clone E7, DSHB, Iowa, USA; diluted 1:10), while anti-5-HT (cat. no. S5545, Sigma-Aldrich, Saint-Louis, USA; diluted 1:100), polyclonal anti-FMRF amide (ab10352, Abcam, Cambridge, UK; diluted 1:1000) and polyclonal anti-synapsin I (ab64581, Abcam; diluted 1:100) were used for tagging the compartments of nervous system. The latter three primary antibodies, having affinity to the nervous system, were first tested together (see below as the “triad”) on several specimens, then also separately. Larvae were incubated with primary antibodies diluted in PBTrX with 5% GS for 4 days at 4 °C. Unbounded antibodies were removed by rinsing in PBTrX three times for 15 min each, and then followed by incubation with secondary antibodies Alexa Fluor 488 and Alexa Fluor 647 (both Thermo Fisher Scientific, Waltham, USA; diluted 1:500 in PBTrX + 5% GS) for 2 days at 4 °C. DAPI staining (Sigma-Aldrich; diluted 1:10,000) was performed for 25 min after three baths of rinsing in PBTrX followed by a last bath in dH_2_O. Before mounting, specimens was rinsed three times in dH_2_O, dehydrated through an ethanol series of increasing concentration (up to 100%) and clarified in methyl salicylate (Sigma-Aldrich). The same medium was used for observation through an Olympus FluoView™ FV1000 confocal microscope (Olympus, Tokyo, Japan) at the Institute of Entomology, Biology Centre of the Czech Academy of Sciences, České Budějovice, Czech Republic (hereafter BC CAS). Confocal images were captured using software Olympus FluoView™ FV10-ASW v.4.2b and processed by Imaris (Bitplane v.6.3.1.).

### Transmission electron microscopy (TEM)

Three live specimens of *D. latus* were cut into suitably small pieces, fixed in cold (4 °C) 1.5% glutaraldehyde and 1.5% paraformaldehyde solutions in 0.1 M Hepes (pH 7.4) and stored at 4 °C. After washing with 0.1 M Hepes (pH 7.4), they were post-fixed in cold (4 °C) 1% osmium tetraoxide (OsO_4_) in the same buffer for 1 h, dehydrated in a graded series of acetone, embedded in Spurr’s epoxy resin and polymerized at 62 °C for 48 h. Ultrathin sections (60–90 nm in thickness) were cut on a Leica Ultracut UCT ultramicrotome (Leica, Wetzlar, Germany), placed on copper grids and stained sequentially with uranyl acetate and lead citrate according to Reynolds (1963). The sections were viewed under a JEOL 1010 transmission electron microscope (JEOL, Tokyo, Japan), equipped with a CCD digital camera Mega View III at 80 kV (Laboratory of Electron Microscopy, Institute of Parasitology, BC CAS).

### Scanning electron microscopy (SEM)

Three saline-rinsed specimens were fixed in hot (almost boiling) 4% formaldehyde solution in order to evert the scolex part. After two weeks, formaldehyde solution was replaced by 70% ethyl alcohol and stored. Subsequently, the material was dehydrated in an ascending series of ethanol (80%, 90%, 96%, 100%; 20 min at each concentration). Chemical drying was performed by hexamethyldisilazane (Sigma-Aldrich) and a JEOL JFC 1300 was used for gold sputtering. Micrographs were made on a JEOL JSM 6510LA (Institute of Parasitology, Slovak Academy of Sciences, Košice, Slovakia) and a JEOL JSEM 7401F (Laboratory of Electron Microscopy, Institute of Parasitology, BC CAS).

## Results

### Immunocytochemistry

**Fig. 1:**
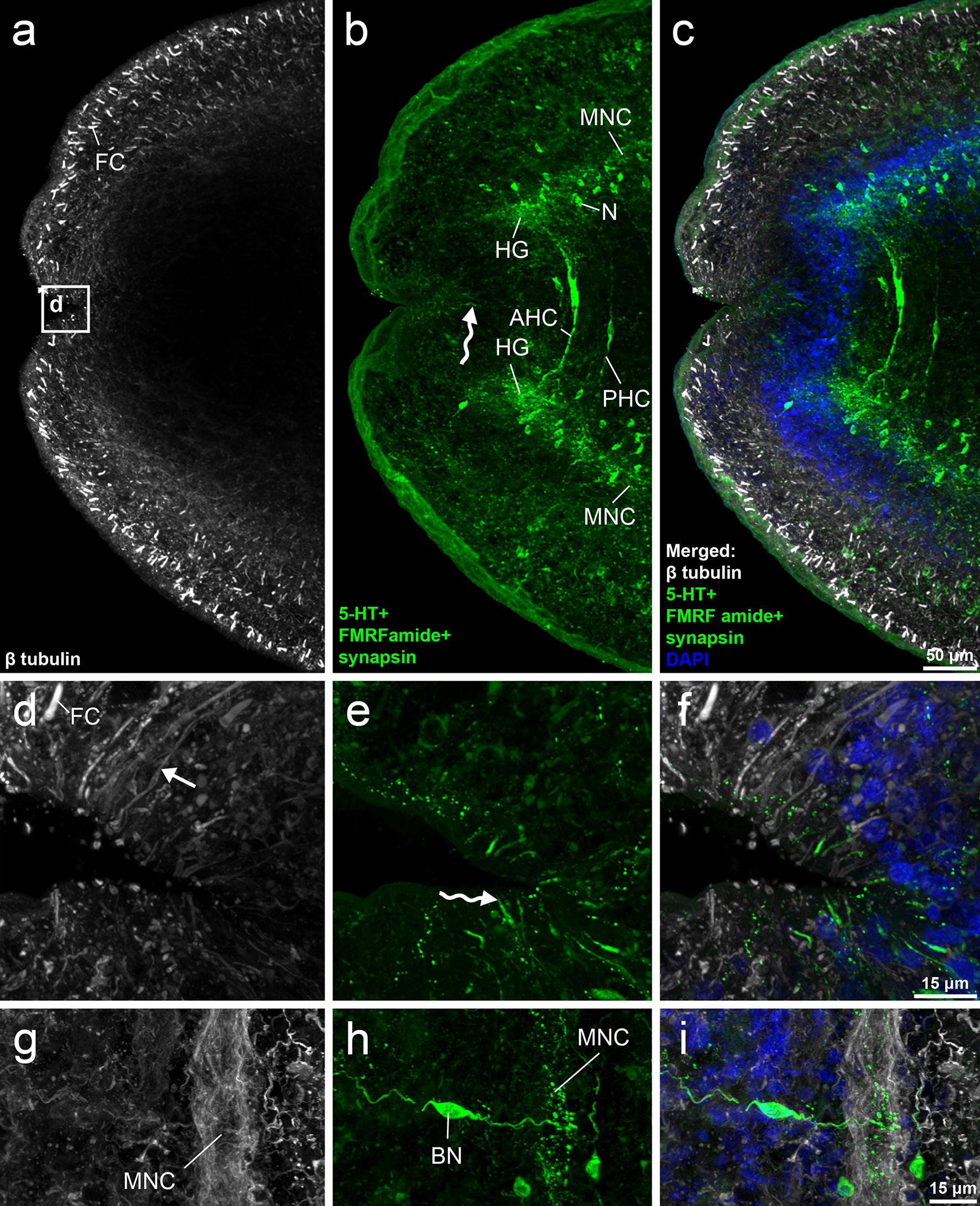
CLSM images of *Dibothriocephalus latus* (conventionally treated plerocercoid). Anterior body part with inverted scolex (**a**–**c**). Detail of apical part with two type of fibres (**d**–**f**). Area of main nerve cord near the posterior end of first third of plerocercoid body with 5-HT-IR bipolar neuron (**g**–**i**). Note solely β tubulin-IR fibres (straight arrows) and second type (sinuous arrows) showing 5-HT-IR and weak β tubulin-IR. *Abbreviations*: AHC, anterior head commissure; BN, bipolar neuron; FC, flame cell; HG, head ganglion; MNC, main nerve cord; N, neuron body; PHC, posterior head commissure

In the conventionally processed *D. latus* plerocercoids, the compartments of the central nervous system (CNS) were labelled by an antibody triad, i.e. anti-5-HT, anti-FMRF amide and anti-synapsin, and the cell tubular system was visualised by the anti-β tubulin antibody. The co-localization of the triad-IR with β tubulin-IR in the CNS was recorded solely in the posterior region of the main nerve cords and associated neurites (Fig. 1g–i). The central nervous system consisted of two head ganglia interconnected with two commissures. The anterior head commissure was more robust and possessed a large bipolar nerve cell body in its middle region, while a weaker commissure with a similar neuron body occurred more posteriorly (Fig. 1b, c). From each head ganglion, a single main nerve cord arose (Fig. 1b) and continued toward the posterior part of the body. Associated nerve cell bodies were present around the head ganglia (Fig. 1b) and surrounded the main nerve cords towards the posterior body end. The bipolar neuron cell body closely associated with main nerve cord was recorded at the end of first third of the body (Fig. 1g–i).

Out of the CNS, a remarkable aggregation of thin, slightly sinuous fibres of two types were observed in the subtegumental parenchyma on the inverted apical part of scolex. The first type was represented by numerous β tubulin positive fibres; their number conspicuously decreased on the lateral sides of the scolex. The fibres of the second type showed both the triad-IR and weak β tubulin-IR and were significantly less numerous than first type (Fig. 1d–f). The flame-cells, compartments of excretory system, were solely β tubulin-IR and occurred more densely in the subtegumental and cortical parenchyma (Fig. 1a, c), than in the medullar parenchyma.

**Fig. 2:**
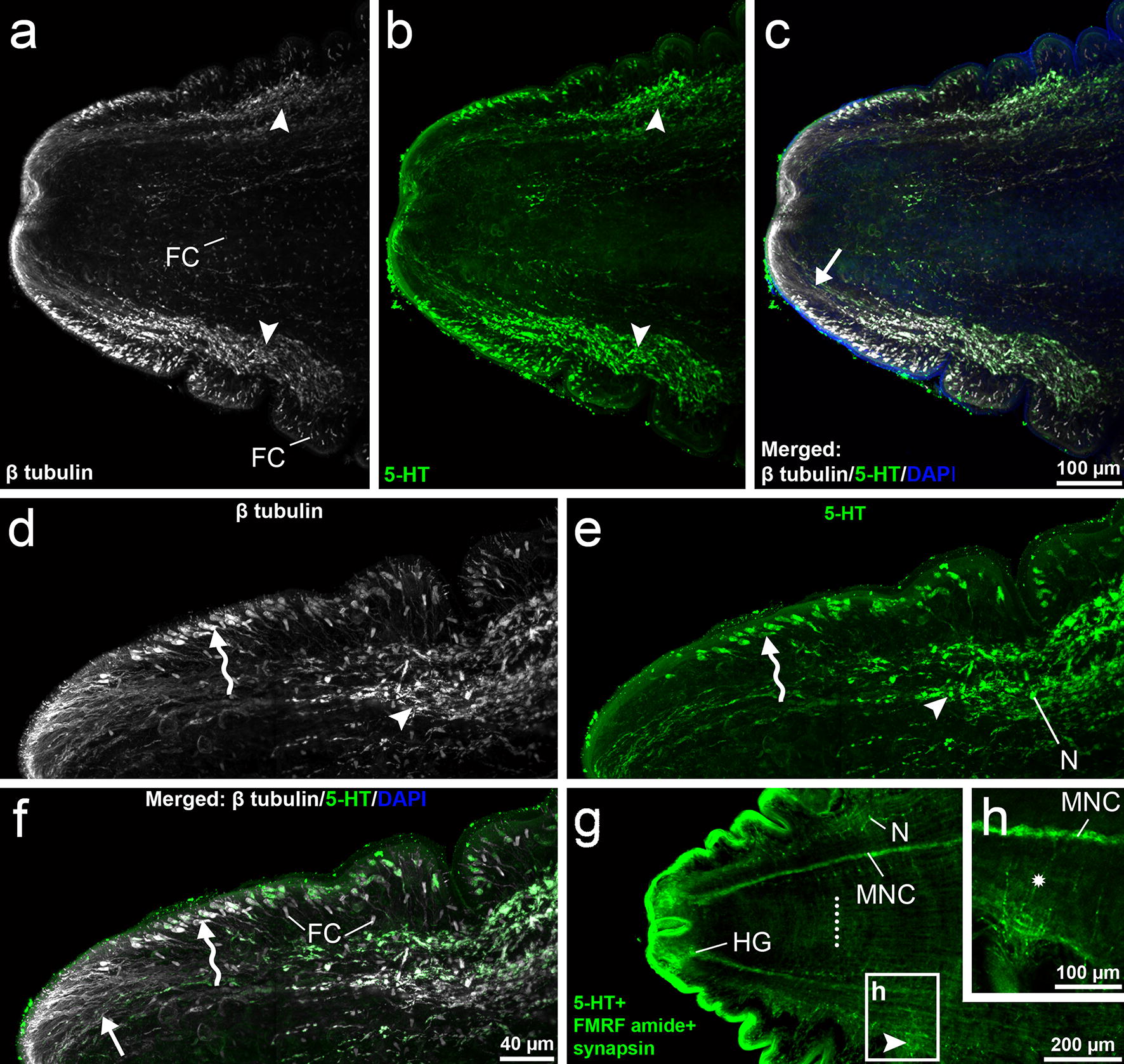
CLSM images of *Dibothriocephalus latus* on the level of CNS (heat-treated plerocercoid). Anterior part of the plerocercoid with everted scolex. Note two nerve plexuses (arrow heads) and the fibres (straight arrow) running towards the tegument (**a**–**c**). Detail of nerve plexus (arrowheads) and adjacent long fibres reaching the tegument on the most apical part of scolex (straight arrows) and shorter fibres with bulky subtegumental nerve cell bodies on the lateral side (sinuous arrows). Note more numerous β tubulin-IR fibres in comparison with 5-HT-IR fibres in apical part of scolex (**d**–**f**). Anterior body part shows position of CNS compartments and nerve plexus (arrowhead); white dots demark posterior margin of bothria (**g**). Detail of transversal commissures (asterisk) connecting main nerve cord with nerve plexus (**h**). *Abbreviations*: FC, flame cell; HG, head ganglion, MNC, main nerve cord; N, neuron body

**Fig. 3:**
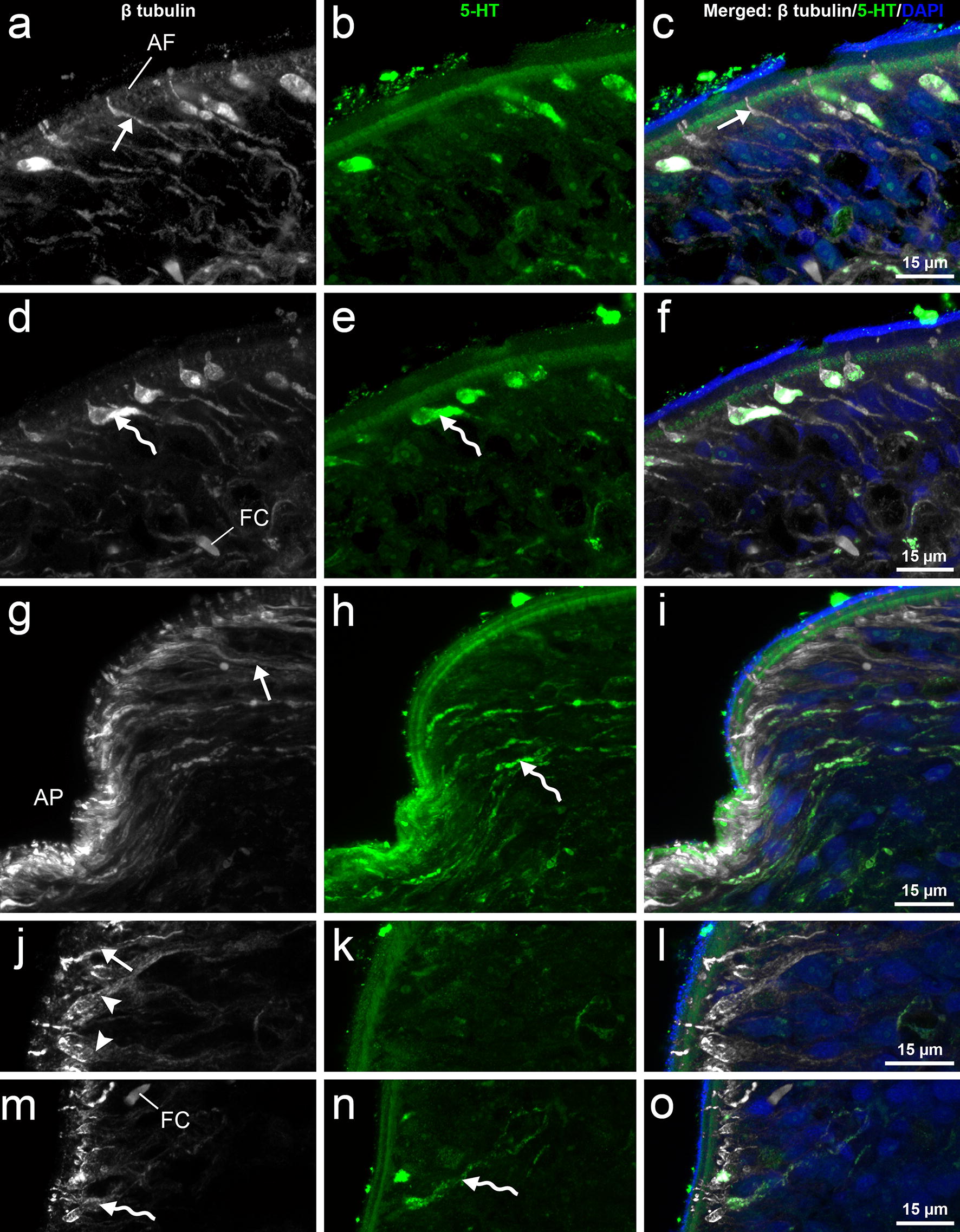
CLSM images of subtegumental structures of *Dibothriocephalus latus* (heat-treated plerocercoids). Detail of lateral scolex part with thin β tubulin-IR fibres (straight arrows) reaching tegument with their terminal parts (**a**–**c**). Detail of lateral scolex part with subtegumental β tubulin-IR and 5-HT-IR bipolar neurons with short projections reaching tegument (sinuous arrows) and their long fibres running toward nerve plexus (**d**–**f**). Apical part of scolex with terminal parts of distinct fibres types. Note numerous β tubulin-IR fibres (straight arrow) and much less frequent type showing co-localisation of β tubulin-IR and 5-HT-IR (sinuous arrow) (**g**–**i**). Terminal parts of three types of fibre structures in most apical part of scolex. Note rare thin β tubulin-IR fibres (straight arrow), wider β tubulin-IR and 5-HT-IR type (sinuous arrows), which both partly resemble fibres on lateral side of the scolex, and finally, numerous wide, solely β tubulin-IR fibre-like structures (arrowheads) (**j**–**o**). *Abbreviations*: AF, anchoring fibril; AP, apical pore; FC, flame cell

**Fig. 4:**
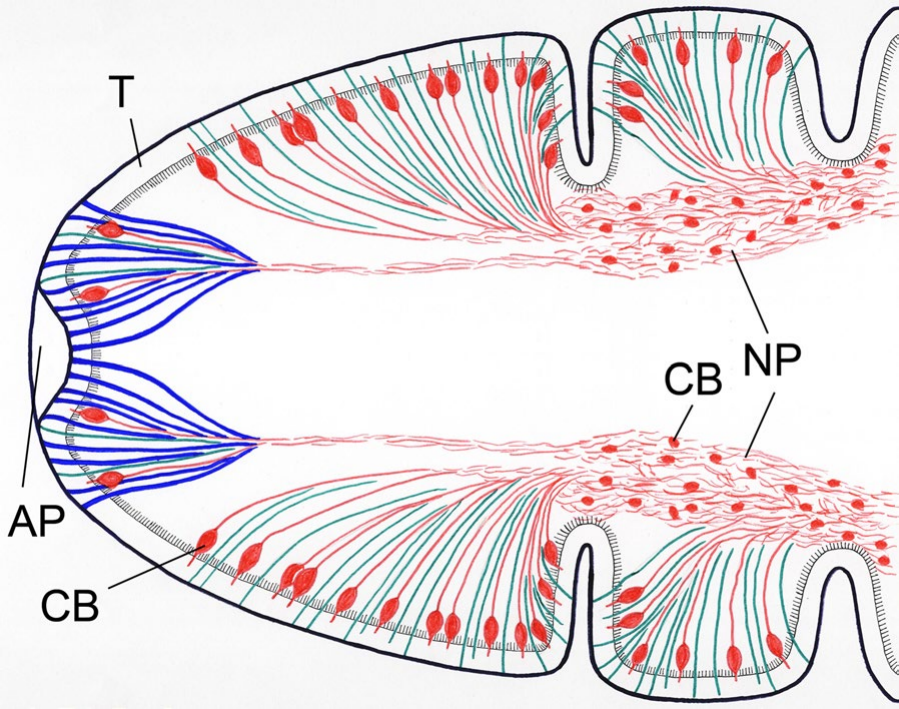
Diagrammatic line drawing of nerve plexuses and adjacent fibre-like structures of *Dibothriocephalus latus* plerocercoid based on CLSM observations. Note 5-HT-IR nerve fibres with large cell bodies under the tegument (red), thin β tubulin-IR nerve fibres (dark green) and wide β tubulin-IR fibre-like structures in apical part of scolex (blue). *Abbreviations*: AP, apical pore; CB, cell bodies; NP, nerve plexus; T, tegument

In heat-treated larvae, two complex nerve plexuses showing co-localization of β tubulin-IR and 5-HT-IR occurred in the cortical parenchyma of the scolex (*n* = 2), each one laterally to the bothria (Figs. 2a–c, 4). These structures did not reach the apical part of the scolex, where the head ganglia were located (Fig. 2g); the plexuses were usually distributed on the level of the middle third and the end of bothria (*n* = 2). From each plexus, several types of fibres arose mostly from the first half of the plexus and ran toward the tegument on the level and anteriorly to the plexus, i.e. on the surface of bothria folds (dorsal, ventral and lateral sides), while a significant bundle of the fibres (Figs. 2d–f, 4) ran toward the anterior scolex margin and reached the area near the apical pore (Fig. 3g–i). The localisation of nerve plexuses and nerve fibres with their terminations can be seen in more detail in Additional file 1: Video S1.

On the surface of bothria folds, two types of fibres from the nerve plexus eached the tegument. The fibres of the first type were β tubulin-IR, thin in their whole course and often penetrate the tegument by their distal part (Figs. 3a–c, 4). The fibres of the second type were both β tubulin-IR and 5-HT-IR, and possessed bulky enlargements (7–14 μm long and 3.5–5 μm wide), i.e. large nerve cell bodies, located under the tegument or in its proximity. From each of these cell bodies, a single short β tubulin-IR projection (rarely also 5-HT-IR) entered the tegument (Figs. 3d–f, 4). In the apical part of the scolex, similar fibres of two types were recorded in the proximity of the apical pore; however, the 5-HT-IR nerve bodies were found to be smaller and more elongate (Figs. 3j–o, 4). Moreover, a third type of a solely β tubulin-IR fibre-like structure with wide terminal (subtegumental) parts and thin intrategumental projections dominated the two above-mentioned fibre types in the apical scolex area (Fig. 3j–h).

The organisation of triad-IR CNS compartments corresponded with that of conventionally treated specimens; however, the position of the head ganglia was more anterior in heat-treated specimens (Fig. 2g). Considering the mutual position of the head ganglia and nerve plexuses, they did not overlap, as the former were located more anterior that the latter, as shown in Fig. 2a–g.

The flame cells were β tubulin-IR and occurred in a similar pattern as in non-heat-treated specimens, i.e. a remarkable number of them were in subtegumental and deeper layers of the cortical parenchyma (Figs. 2a, f; 3d, m).

### Ultrastructural observations

#### Surface ultrastructure

**Fig. 5:**
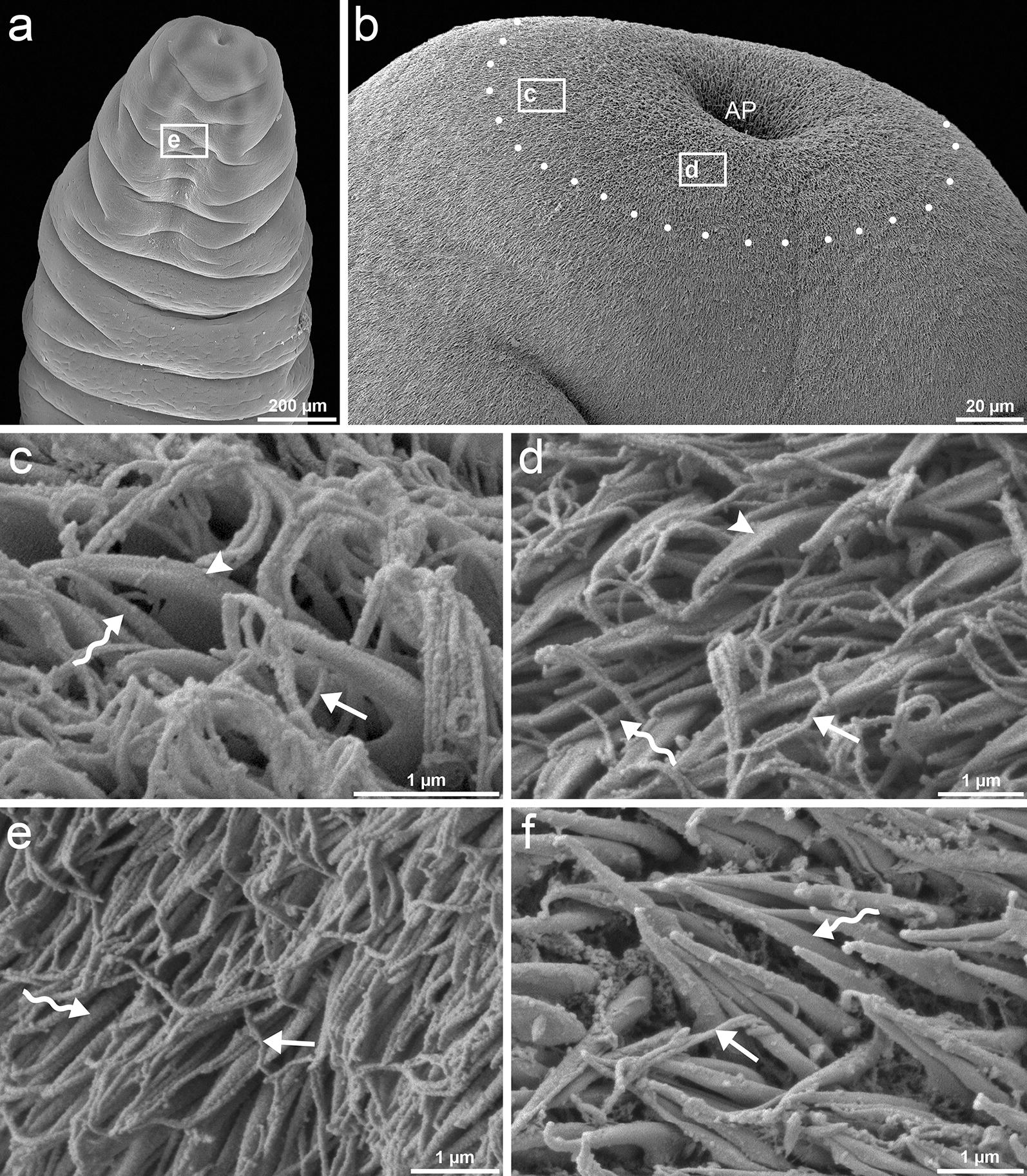
SEM micrographs of *Dibothriocephalus latus* plerocercoids surface (hot fixation). Anterior part of the plerocercoid showing everted scolex with bothria (**a**). Apical part of scolex; approximate area with observable uncinate spinitriches demarked by white spots (**b**). Apical region in detail (**c**, **d**). Inner surface of bothria (**e**). Middle part of the plerocercoid (**f**). Note three different types of microtriches, i.e. uncinate spinitriches (arrow heads), coniform spinitriches (sinuous arrows) and capilliform filitriches (straight arrows) (**a**–**f**). *Abbreviation*: AP, apical pore

**Fig. 6:**
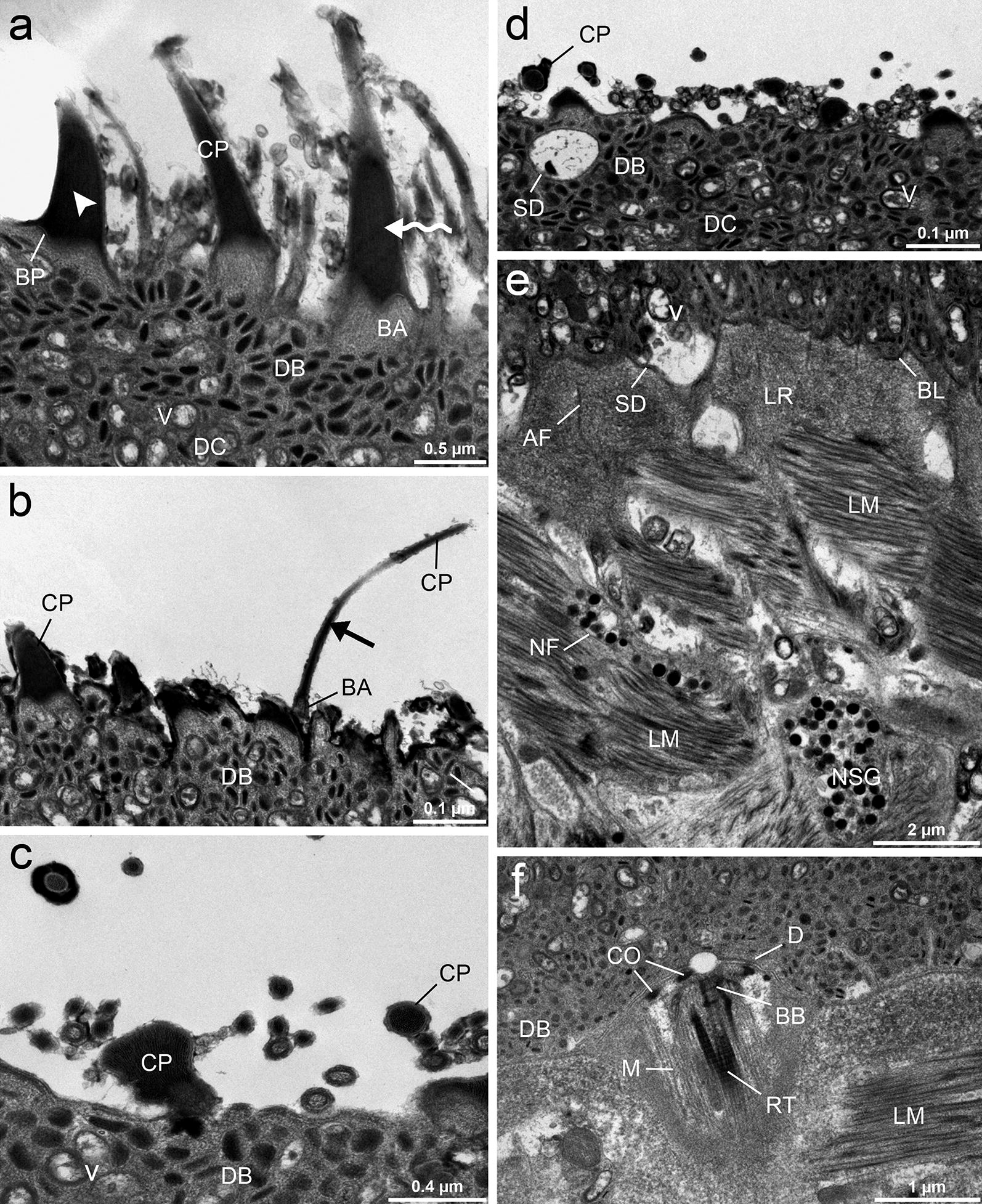
TEM micrographs of surface ultrastructure of *Dibothriocephalus latus* plerocercoids. Uncinate (arrowhead) and coniform (sinuous arrow) spinitriches and solitary distributed long capilliform filitriches (straight arrow); tegument composed of a distal cytoplasm containing numerous electron-dense bodies and vesicles (**a**, **b**). Cross-sections through the two types of microtriches. Note the structure of the coniform spinitriches cap (**c**). The distal cytoplasm with electron-dense bodies, vesicles and the secretory duct after discharge of the secretory granules (**d**). Underlying perikariya. The basal lamina delimited the distal cytoplasm from the underlying longitudinal and circular muscle layers. The lamina reticularis is filled with anchoring fibrils, oriented perpendicularly to the basal membrane (**e**). Longitudinal section through a non-ciliated receptor. Note the presence of four dense collars, basal body, striated rootlets and a bundle of microtubules (**f**). *Abbreviations*: AF, anchoring fibrils; BA, base; BP, baseplate; BB, basal body; BL, basal lamina; CO, collars; CP, cap; D, desmosomes; DB, dense bodies; DC, distal cytoplasm; LM, longitudinal muscles; LR, lamina reticularis; M, microtubules; NF, nerve fibre; NSG, neurosecretory granules; RT, striated rootlets; SD, secretory duct; V, vacuoles

Three types of microtriches were observed on the scolex and body surface of *D. latus* plerocercoids, namely (i) hook-shaped uncinate spinitriches with a very wide base and robust and posteriorly curved cap (Figs. 5c, d; 6a); (ii) coniform spinitriches whose straight cap merges with a slightly wider base (Figs. 5c–f; 6a, b); and (iii) capilliform filitriches with a slim base and a long, thin and often sinuous cap (Figs. 5c–f; 6b, c). In all three types of microtriches, the proximal base was separated from the distal electron-dense cap by a transverse base plate composed of two dense layers separated by a more electron-lucent layer (Fig. 6a). The base of the coniform spinitriches contained tegumental distal cytoplasm with longitudinal filaments and was surrounded by an electron-dense tunic (Fig. 6a). Cross-sections through the microtriches cap showed that it consists of an electron-dense medulla enclosed by a less dense cortex. The dense medulla contained a number of tubular microfilaments that run longitudinally throughout the cap (Fig. 6c, d). The three types of microtriches were not distributed in the same manner on the body surface. The uncinate spinitriches (~ 2 µm long) were recorded only on the most apical part of the scolex (Fig. 5a–d). The coniform spinitriches (~ 2 µm long) were unevenly distributed on the entire body tegument; they were the most numerous type of microtriches on the body surface, except for the apical part of the scolex (Fig. 5c, d, f). The capilliform filitriches (~ 3 long) were also observed on whole body surface and appeared to be more numerous on the tegument of apex and bothria (Fig. 5c–e), while otherwise were sparsely distributed (Fig. 5f).

The syncytial tegument was composed of a thick distal cytoplasm and underlying perikarya. TEM examination of the distal cytoplasmic layer revealed organelles represented by different types of inclusions, referred to herein as electron-dense discoidal bodies oriented perpendicular to the surface, electron-dense spherical bodies concentrated toward the apical plasma membrane and numerous vesicles, some of which were empty and the others contained small granules (Fig. 6a, b). The distal cytoplasm was attached to the sunken perikarya by a basal lamina. The basal lamina consisted of two layers, the outermost dense layer (lamina dense) and the inner layer of a fibrillar extracellular lamina (lamina reticularis). Bands of longitudinal and circular muscles lay beneath the distal cytoplasm. At the ultrastructural level, radial electron-dense fibrils, oriented perpendicularly to the basal lamina of the tegument were observed. These anchoring fibrils acted as a specialized connective tissue structures that interconnected the tegument with the subtegumental muscle fibres (Fig. 6e).

Another structure observed between the distal cytoplasm of tegument and the underlying musculature was the non-ciliate sensory receptor with no contact to the outside. The bulb of the receptor was connected to the distal cytoplasm by septate desmosomes and contained a basal body and a large striated rootlet, a bundle of microtubules oriented parallel to the longitudinal axis of the bulb and four dense collars in the apical part of the bulb (Fig. 6f). No receptor-like structures were observed using SEM.

#### Nerve cells

**Fig. 7:**
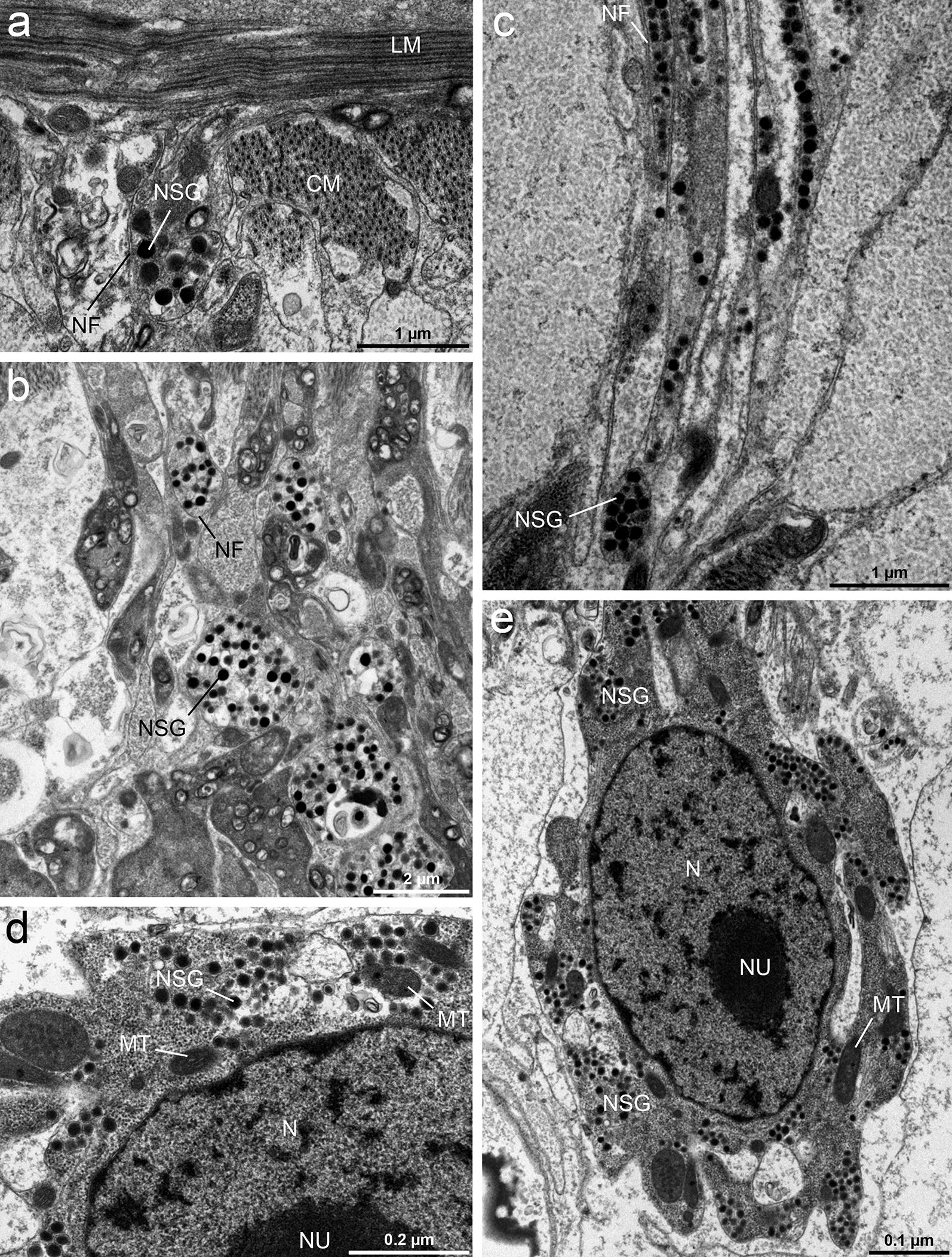
TEM micrographs of nerve cells of *Dibothriocephalus latus* plerocercoids. Section through a nerve fibre under the basal lamina and longitudinal musculature (**a**). Nerve fibres containing spherical electron-dense granules below the muscle layers (**b**). Longitudinal section through a deeper part of the parenchyma showing nerve fibres with neurosecretory granules (**c**). Neurosecretory granules and mitochondria in the cytoplasm of the nerve cells (**d**, **e**). *Abbreviations*: CM, circular muscles; MT, mitochondria; N, nucleus; NU, nucleolus; BF, nerve fibre; NSG, neurosecretory granules

Nerve cells (approximately 0.5 µm in diameter) were found in the parenchyma throughout the length of the body. At the ultrastructural level, the cells described here usually had an irregular shape with projecting nerve fibres (Fig. 7a–c) and were characteristic by having a high nucleo-cytoplasmic ratio. A large nucleus occupied the central region of the cell and exhibited a distinct nucleolus and numerous small clumps of heterochromatin dispersed in the nucleoplasm (Fig. 7e). The cytoplasm was packed with many free ribosomes and large mitochondria. In addition, large electron-dense granules (100–120 nm in diameter) were abundant in the cytoplasm of the nerve cells and nerve fibres (Fig. 7b–e).

#### Gland apparatus

**Fig. 8:**
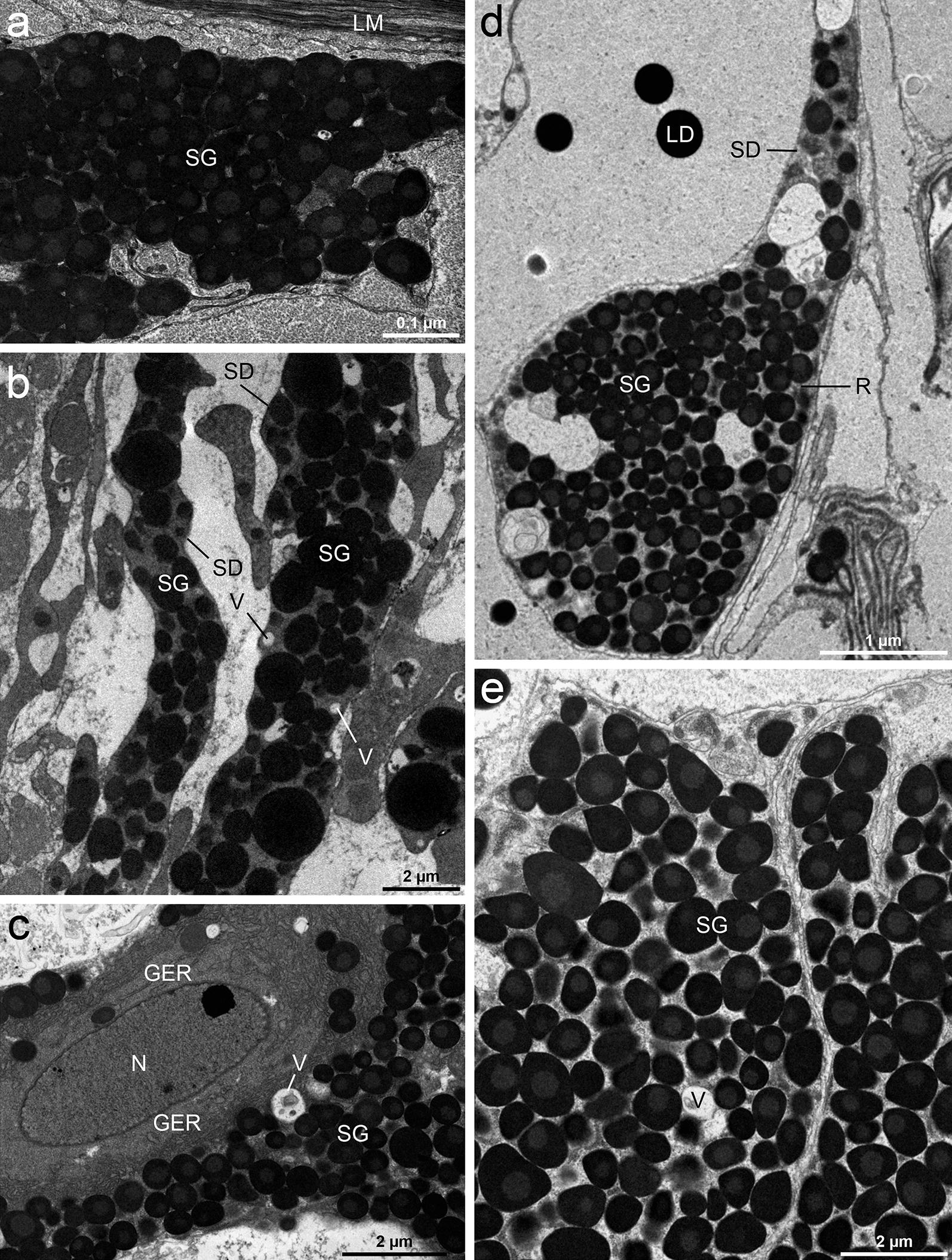
TEM micrographs of *Dibothriocephalus latus* plerocercoids depicting gland-cells and their ducts. Secretory ducts of the gland-cell going towards the distal cytoplasm (**a**, **b**). The nucleus and cytoplasm of gland-cell with extensive granular endoplasmic reticulum, secretory granules and vacuoles (**c**). A longitudinal section through the ducts filled with secretory material. Note a large reservoir formed from dilatation of the duct with great numbers of secretory granules (**d**). Detail of the secretory granules; note their moderately-dense cores (**e**). *Abbreviations*: GER, granular endoplasmic reticulum; LD, lipid droplets; LM, longitudinal muscles; N, nucleus; SD, secretory duct; SG, secretory granules; R, reservoir; V, vacuoles

The gland complex was well developed and fills the parenchyma all along the plerocercoid’s body. The gland-cells possessed irregular cell bodies and relatively long ducts, which connected them with the tegument (Fig. 8a, b). The gland contained a large nucleus, mitochondria and extensive cisternae of granular endoplasmic reticulum (Fig. 8c). Before the ducts passed between the muscles beneath the tegument, they became dilated forming large reservoirs with a great number of secretory granules (Fig. 8d). The secretory granules (*c.* 1000–1200 nm in diameter) were shown to be composed of moderately-dense core being surrounded by more electron-dense layer (Fig. 8e). In addition to the characteristic secretory granules, the cytoplasmic projections of the cell were packed with a number of electron-lucid vesicles (Fig. 8c–e).

#### Excretory system

**Fig. 9:**
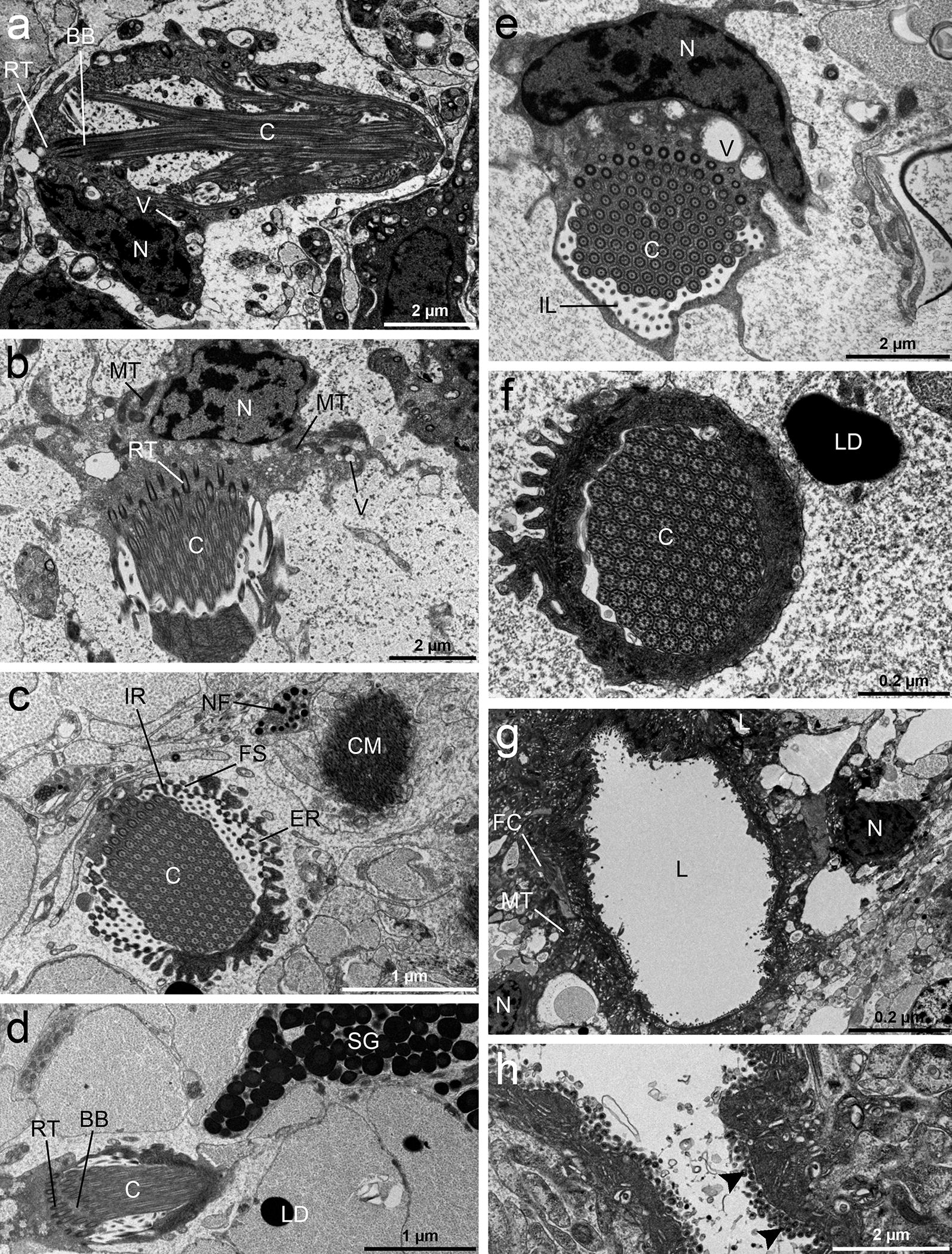
TEM micrographs of flame cells and excretory ducts of *Dibothriocephalus latus* plerocercoids. Longitudinal section of a flame cell showing the large nucleus with several heterochromatic regions, cytoplasmic region with vesicles and mitochondria and “flame” of cilium (**a**). Section through the flame cell showing the cell body attached to the flame through striated rootlets (**b**). Cross-section though the flame cell showing internal ribs of flame cell origin and external ribs of duct cell origin. Ribs are connected by a fibrous sheet (**c**). Section through the flame cell showing cilia with basal bodies and striated rootlets (**d**). Cross-section showing internal leptotriches arising from the flame cell situated between the cilia and ribs (**e**). Cross-section through the “flame” of a flame cell showing the cilia with their typical 9 + 2 pattern of microtubules (**f**). Sections of an excretory duct cell with the nucleus and the lumen and surrounding cytoplasm containing mitochondria and numerous vesicles, some of which may contain electron-dense material (**g**). The inner surface of the ducts is expanded by numerous microvilli (arrowhead) (**h**). *Abbreviations*: BB, basal body; C, cilia; CM, circular muscles; ER, external ribs; FC, flame cell; FS, fibrous sheet; IL, internal leptotriches; IR, internal ribs; L, lumen; LD, lipid droplets; MT, mitochondria; N, nucleus; NU, nucleolus; NF, nerve fibre; SG, secretory granules; V, vesicles; RT, rootlets

The excretory system was composed of variously sized ducts which connected the flame cells to the major collecting ducts. The flame cells exhibited a large body enclosing a tuft of cilia. The base of the flame cell contained a region of tightly packed ciliary rootlets and a single nucleus with several heterochromatin regions (Fig. 9a, b, d). A bunch of long, densely packed cilia with 9 + 2 axonemes was seen inside the ciliary tuft (Fig. 9e). The number of cilia anchored in the cytoplasm by basal bodies possessing striated rootlets reached between 80–100 cilia, and all were aligned in the same direction (Fig. 9c, e, f). A few mitochondria, numerous free ribosomes and some vesicles, empty or filled with electron-dense material were also present in the scant cytoplasm of the cell (Fig. 9a, b, e). The flame and duct cells were connected by interdigitating ribs of cytoplasm, which were separated by a fibrous sheet. The ribs occurring internally to the sheet originated from the flame cell and those occurring externally left the duct cell (Fig. 9c). There were elongate projections of cytoplasm, leptotriches on the inner ribs (Fig. 9c). The walls of all excretory ducts of the protonephridial system were syncytial, consisting of a cytoplasm and underlying nucleated cytons (Fig. 9g). The inner surface of the ducts was covered by microvilli which increased its surface (Fig. 9g, h). The nucleus of a duct cell was localised distally to the lumen and was surrounded by a layer of cytoplasm. The perinuclear cytoplasm contained many mitochondria and numerous vesicles, electron-lucent or filled with electron-dense material (Fig. 9g).

## Discussion

### Surface ultrastructure

The structure of the tegument is similar in adults and plerocercoids of *D. latus* and other diphyllobothriids, with an anucleate distal cytoplasm containing discoidal bodies and vesicles [16, 17]. However, the microtriches pattern of larvae is different from adults of *D. latus*. The surface of the adults is only covered by uniform capilliform filitriches, except for the cirrus and genital atrium, where coniform spinitriches were observed [17, 18]. Our data suggest that coniform spinitriches dominate over interspersed capilliform filitriches and apically-localised massive uncinate spinitriches in plerocercoids which is consistent with Kuperman’s [26] two types of microtriches on the anterior end of *D. latus* plerocercoids. Among other diphyllobothriidean tapeworms, the capilliform filitriches are the most common type reported on larvae as well as adults [17, 19–24], while coniform spinitriches are a much less reported type, e.g. in adults of *Ligula intestinalis* (Linnaeus, 1758), *Diphyllobothrium lanceolatum* (Krabbe, 1865) and on cirrus of *D. latus* [17, 20]. Uncinate spinitriches were observed on the cirrus of *Matticestus anneae* Caira, Jensen & Fyler, 2018 (Onchoproteocephalidae) [25] and on the apical part of plerocercoids (but not in adults) of *Diphyllobothrium tetrapterum* (von Siebold, 1848) by Hernández-Orts et al. [24]. We can speculate which types were captured by Grammeltvedt [26], Kuperman [27] and Mustafina & Biserova [28] on plerocercoids of *D. latus*, *D. dendriticus* and *Pyramicocephalus phocarum* (Fabricius, 1780). They have been reported as “thorn-shaped microvillus”, “claviform-shaped” and “spinthrix microtriches”; however, “claviform” shape could be result of cross-section through massive cap and relatively thin base of uncinate spinitriches.

Such polymorphism of microtriches on larval tapeworms may reflect the functional differences of body regions where they occur [29, 30]. A nutritional function is commonly ascribed to filitriches, which have a large surface area suitable for the uptake of nutrients [31, 32], but anchoring properties can also be inferred [33, 34]. It has been suggested that the spinitriches with a massive wide base are involved in diverse functions, such as locomotion and attachment [33, 35, 36]. Thus, uncinate spinitriches of *D. latus* plerocercoids may facilitate penetration of the intestinal wall and motility in muscles of intermediate paratenic fish hosts, and then help to attach the parasite in its definitive host during the early stage of infection, when the fully functional bothria are not yet developed.

The non-ciliated sensory receptors recorded by TEM in the distal cytoplasm were the only type of receptor found in our specimens. The only available data about sensory receptors in adult *D. latus* are those of Poddubnaya [18], who observed sensory endings near the genital atrium. Non-ciliated receptors were previously described by Okino & Hatsushika [21] in adults of *Spirometra erinaceieuropaei* (Rudolphi, 1819), who attributed them a role during cross-insemination, while Andersen [37] considered receptors without a cilium to be mechanoreceptors or proprioreceptors. Heterogeneous populations of both ciliated and non-ciliated receptors were detected in plerocercoids of *D. ditremus*, *D. dendriticus* and *P. phocarum* (all Diphyllobothriidea) [11, 28, 38]. Moreover, Kutyrev et al. [11] also assumed their secretory function, which includes production of prostaglandin (PGE_2_) as a growth factor, neuroactive compound and/or immunomodulator.

### Nervous system

The morphology of CNS generally corresponds with previously reported observations summarised by Wardle & McLeod [39]. However, our finding of two interganglionic commissures is not congruent with previous observations of a single commissure in the congeneric species, *D. dendriticus* [9, 10]. This observation suggests that the microanatomy of the head ganglia in these tapeworms may be similarly complex as is in the trypanorhynch *Grillotia* spp. (both plerocercoids and adults) possessing one strong posterior and two weaker anterior (dorsal and ventral) commissures [40, 41].

In the scolex of heat-treated larvae, the transversal nerve fibres leave the main nerve cords, run towards lateral body side and meet the nerve plexuses (Fig. 2g, h). Then, subtegumental serotoninergic large nerve cell bodies with short intrategumental projections are connected with the nerve plexus by their long neurites. The highest number of these sensory organs was observed on the surface of bothria folds, some of them inside the bothria, while relatively few of them with a slightly different shape occurred near the apical pore. A similar situation was recorded considering thin, solely β tubulin-IR fibres; however, the chemical nature of their neurotransmitters remains unknown. The third type, wide and β tubulin-IR structures, could represent the terminal part of frontal gland-cell ducts (see below). Nerve plexuses and subtegumental serotoninergic cell bodies were not observed in conventionally treated plerocercoids (*n* = 2).

Serotoninergic structures of both CNS and PNS were recorded within several flatworm taxa including the subsurface nerve net and sensory organs [42, 43] and serotonin was reported as an excitatory neurotransmitter [44]. In the congeneric species *D. dendriticus*, the serotoninergic plexus was identified on the outer surface of the longitudinal muscles and 5-HT-IR nerve fibres innervated bothria folds [45].

Considering ultrastructure, the most striking feature of the nerve cells and the nerve fibres extending from these cells is the presence of a large number of electron-dense neurosecretory granules (*c.* 100 nm), regarded as peptidergic-type of granules (see Gustafsson [46]).

### Gland apparatus

In the present study, a well-developed gland complex was recognised in the cortical and the medullary parenchyma of the scolex and the whole body by TEM. The ducts of gland-cells possessed very large granules with a moderately-dense core and their plasma membranes should be reinforced by longitudinally oriented microtubules [14, 47]. By CLSM, three types of β tubulin-IR fibre-like structures were captured in the apical part of the scolex. Two of them were identified as nerve fibres (see above), while the function of third type remained questionable. Considering the anticipated microtubular armature of the frontal gland ducts of diphyllobothriids and reservoir-like shape of their terminal parts [6, 14, 48], this third β tubulin-IR type of structure could represent the terminal ducts of gland apparatus, which release their secretory granules near the apical pore of scolex. This assumption is also supported by observations on plerocercoids of related species, which showed intimate localization of sensory endings and terminal parts of gland duct in *Pyramicocephalus phocarus*, and accumulation of gland duct terminals near the “frontal pit” in *Spirometra erinacei* [28, 49].

The occurrence of a gland apparatus in plerocercoids of *Dibothriocephalus* spp. has been pointed out by a number of authors and used as a taxonomic characteristic in their systematics [14, 48, 50, 51]. Its fine structure was found to be quite similar among species of this genus, although *D. latus* possess the largest gland apparatus in comparison with *D. dendriticus*, *D. ditremus* and *D. nihonkaiensis* [14]. The cytoplasm of the gland-cells contains a strongly developed granular endoplasmic reticulum, mitochondria, ribosomes and secretory granules suggesting active protein synthesis in these cells. The secretory granules go through the ducts into the distal cytoplasm of the tegument [47, 52]. The discharge of the secretion by plerocercoids and adults of *D. latus* into the environment occurs according to an eccrine mode of secretion, i.e. through the ducts penetrating the tegument directly to the environment [53]. In the terminal parts the ducts, reservoirs similar to those reported herein were observed in *D. dendriticus*, *D. ditremus* and *D. latus* [6, 14, 48]. Two types of frontal gland-cells (“green” and “golden”) were distinguished in plerocercoids of *D. dendriticus* [54]. During the first day of *in vitro* and *in vivo* cultivation, “green glands” significantly increased the production of their secretion, which exhibited adhesive character *in vitro*. Thus, it may play a role during the early establishment of the parasite in the definitive host’s intestine. After few days of cultivation, this sulphur-rich type of cells completely disappeared, corresponding to a weakly developed gland system in adult *D. latus* [53].

The chemical nature of the gland secretion is poorly known in most tapeworms. The cytoplasm of gland-cells of *D. dendriticus* plerocercoids was weakly positive for carbohydrate-protein complexes, unlike the secretory granules inside the ducts of these cells, which were generally unreactive to cytochemical tests [6]. In the same species, lipid immunomodulator prostaglandin PGE_2_ was identified as product of frontal gland-cells [45]. The frontal glands (also called “penetration glands”) have usually been connected to either penetration or migration in intermediate host, or the production of adhesive substances in the definitive host [6, 14, 37, 53–55]. The role of frontal glands during the penetration of *Dibothriocephalus* spp. plerocercoids in intermediate hosts was initially proposed by Kulow [55], who assumed that their secretory products might soften the intestinal tissues of piscivorous fishes when infected by their prey. This assumption was experimentally supported by Kuperman & Davydov [14] on *D. latus* passing through the stomach wall of the northern pike, *Esox lucius.* Ӧhman-James [6], however, inclined to believe in adhesive nature of this frontal gland secretion as the cytochemical tests on *D. ditremus* were negative. More recently, cystein and serin proteases were identified as fundamental secretory/excretory products for the invasion of *S. erinaceieuropei* plerocercoids in its intermediate host [56–58].

### Excretory system

Although the structure of the excretory system of cestodes has been studied by many investigators [59–65], its function has not yet been completely defined. The protonephridial system of *D. latus* plerocercoids shows an organization very similar to that described in adult *D. latus* by Von Bonsdorff & Telkkä [62]. It is made up of two main components, i.e. two pairs of excretory ducts and flame cells. It has been suggested that the flame cells accomplish excretory activity and take a role in the maintenance of the osmoregulation of cestodes within their hosts. Furthermore, the number of axonemes of the cilia of the flame could be useful for phylogenetic considerations as proposed for the phylum Platyhelminthes [27, 66, 67]. It has been postulated that the continuous beating of the cilia of the flame cells draws waste products to the outside through the epithelial cells of the duct walls, thus providing a possible filtration system for the body fluids. Kutyrev et al. [11] found that flame cells of the excretory system are involved in the metabolism of prostaglandins in *D. dendriticus* and thus may participate in the excretion of prostaglandins (possible immunomodulator) into host tissue. In our specimens, they were solely β tubulin-IR due to the presence of axonemes and a DAPI-positively stained nucleus (Fig. 8i). The flame cells are joined with the ducts by the interdigitation of ribs in the cytoplasm, which are connected to each other by a fibrous sheet. The epithelium lining the excretory ducts has a structure which suggests that it is metabolically active. The luminal surface of the excretory ducts is significantly increased due to numerous microvilli, cytoplasmic projections and folds which suggest their role in the reabsorption of filtrated material [68].

### The benefits of heat-treatment

The heat treatment prior to formalin fixation for CLSM (also used by Rozario & Newmark [8]) seems to have a beneficial effect on visualizing of particular nervous system-related structures of *D. latus* plerocercoids. Indeed, heat-mediated antigen retrieval is a common signal increasing procedure in IF-based techniques; however, it used to be applied after formalin fixation [69]. In our case, heat treatment was used prior the fixation and might act as “protective agent” against the formalin effect and thus preserve the structure of antigens and/or make the parenchyma of larvae more penetrable for antibodies.

## Conclusions

Both confocal and electron microscopy together provided novel data about *D. latus* plerocercoids, i.e. depicted the complexity of the nervous system and frontal glands, captured three different types of microtriches and revealed the presence of a single type of sensory receptor. As well-developed gland complex and the presence of the spinitriches were not observed in adult *D. latus*, these features are probably relevant for plerocercoids and may facilitate their surviving in intermediate fish hosts and possibly also conduce to attachment of the tapeworm in the definitive hosts.

## Additional file

**Additional file 1: Video S1.** A movie showing distribution of both superficial and internal structures in the scolex of *D. latus* plerocercoids. Performed on the same individual shown in Fig. 2a–f; β tubulin-IR structures are white, serotonin-IR structures are green and DAPI staining displays in blue.

## Data Availability

All data supporting the conclusions of this article are included within the article and its additional file.

## Abbreviations

SEM: scanning electron microscopy
TEM: transmission electron microscopy
CLSM: confocal laser scanning microscopy
IR: immunoreactive
5-HT: 5-hydroxytryptamin
PBS: phosphate-buffered saline
PB: phosphate buffer
PBTrX: phosphate buffer with Triton X
FMRF amide: phenylalanyl-l-methionyl-l-arginyl-l-phenylalanin amide
DSHB: Developmental Studies Hybridoma Bank
GS: goat serum
DAPI: 4′,6-diamidino-2-phenylindole
Hepes: hydroxyethyl piperazineethanesulfonic acid
BC CAS: Biology Centre, Czech Academy of Sciences
CNS: central nervous system
PNS: peripheral nervous system
PGE_2_: prostaglandin E_2_
